# Branched Chain Amino Acids Promote ATP Production Via Translocation of Glucose Transporters

**DOI:** 10.1167/iovs.63.9.7

**Published:** 2022-08-05

**Authors:** Sachiko Iwai, Tomoko Hasegawa, Hanako Ohashi Ikeda, Akitaka Tsujikawa

**Affiliations:** 1 Department of Ophthalmology and Visual Sciences, Kyoto University Graduate School of Medicine, Kyoto, Japan; 2 Research Fellow of the Japan Society for the Promotion of Science, Tokyo, Japan

**Keywords:** branched chain amino acid, neuroprotection, ATP, glucose uptake, glucose transporters

## Abstract

**Purpose:**

We have previously shown that maintenance of ATP levels is a promising strategy for preventing neuronal cell death, and that branched chain amino acids (BCAAs) enhanced cellular ATP levels in cultured cells and antagonized cell death*.* BCAAs attenuated photoreceptor degeneration and retinal ganglion cell death in rodent models of retinal degeneration or glaucoma. This study aimed to elucidate the mechanisms through which BCAAs enhance ATP production.

**Methods:**

Intracellular ATP concentration was measured in HeLa cells under glycolysis and citric acid cycle inhibited conditions. Next, glucose uptake was quantified in HeLa cells and in 661W retinal photoreceptor-derived cells under glycolysis inhibition, endoplasmic reticulum stress, and glucose transporters (GLUTs) inhibited conditions, by measuring the fluorescence of fluorescently labeled deoxy-glucose analog using flow cytometry. Then, the intracellular behavior of GLUT1 and GLUT3 were observed in HeLa or 661W cells transfected with enhanced green fluorescent protein-GLUTs.

**Results:**

BCAAs recovered intracellular ATP levels during glycolysis inhibition and during citric acid cycle inhibition. BCAAs significantly increased glucose uptake and recovered decreased glucose uptake induced by endoplasmic reticulum stress or glycolysis inhibition. However, BCAAs were unable to increase intracellular ATP levels or glucose uptake when GLUTs were inhibited. Fluorescence microscopy revealed that supplementation of BCAAs enhanced the translocation of GLUTs proteins to the plasma membrane over time.

**Conclusions:**

BCAAs increase ATP production by promoting glucose uptake through promotion of glucose transporters translocation to the plasma membrane. These results may help expand the clinical application of BCAAs in retinal neurodegenerative diseases, such as glaucoma and retinal degeneration.

Neuronal cell death causes incurable eye diseases, such as retinal degenerative diseases and glaucoma, which are major causes of blindness worldwide. Among retinal degenerative diseases, retinitis pigmentosa is the most common type of hereditary retinal degeneration, and is characterized by progressive damage to photoreceptors. Although various potential therapeutic strategies are currently being investigated^1^^–^^5^ and several clinical trials are ongoing,^6^ therapies that prevent photoreceptor cell death have not been established. For glaucoma, intraocular pressure reduction is the only established effective strategy to prevent retinal ganglion cell death; however, there are considerable cases in which visual impairment progresses even when the intraocular pressure is reduced.^7^

We have previously shown that the maintenance of intracellular ATP levels antagonizes cell death. Prevention of ATP consumption by inhibiting the ATPase activity of valosin-containing protein, the most abundant soluble ATPase within a living organism, prevented neuronal cell death in glaucoma,^8^ retinal degeneration,^9^^–^^11^ and retinal artery occlusion.^12^^,^^13^

Another way to maintain intracellular ATP levels is by enhancing ATP production. We have previously shown that supplementation with branched-chain amino acids (BCAAs) increased intracellular ATP levels and protected cultured cells against endoplasmic reticulum (ER) stress and mitochondrial respiratory chain inhibition stress.^14^^,^^15^ Additionally, we have shown that supplementation with BCAAs attenuated photoreceptor cell death in mouse models of retinitis pigmentosa, and attenuated retinal ganglion cell death in a mouse model of glaucoma.^14^

BCAAs are important energy sources. Degraded BCAAs are used as energy sources in various proportions in the citric acid cycle, depending on the cell type or conditions.^16^^–^^18^ BCAAs have also been reported to improve glucose metabolism in cirrhotic liver,^19^ and promote glucose uptake in skeletal muscle in rodents.^20^^–^^22^ However, the mechanisms through which BCAAs promote glucose metabolism have not been fully elucidated. Clinically, BCAAs have been used to treat patients with liver cirrhosis, and have been shown to reduce the incidence of complications.^23^^–^^25^

In the current study, we elucidated the mechanisms through which BCAAs enhance ATP production.

## Methods

### Cell Culture and ATP Measurement

HeLa cells were cultured in Dulbecco's modified Eagle's medium (DMEM) containing 4.5 g/L of glucose and 0.1% fetal bovine serum without amino acids (Wako Pure Chemical Industries, Ltd., Osaka, Japan, 048-33575). BCAAs (L-isoleucine: L-leucine: L-valine = 1:2:1.2, the same as LIVACT; Ajinomoto Co., Tokyo, Japan) were added to the medium (0.04, 4.0, or 40 mM, molecular weight was calculated as 126.829 g/mol from the respective molecular weights and the abundance ratio in the formulation).^14^ Glycolysis inhibition was induced by a hexokinase inhibitor, lonidamine (1-(2,4-Dichlorobenzyl)-1H-indazole-3-carboxylic acid, 300 µM, [used in a previous study at 150 µM for HeLa cells,^26^ the same concentration as shown in Supplementary Fig. S1A], Abcam, Cambridge, UK, ab142442); a glyceraldehyde 3-phosphate dehydrogenase (GAPDH) inhibitor, heptelidic acid (0.6 µM [used in a previous study at 35 µM for various cancer cell lines^27^; at this concentration, we observed excessive cell death in the current study], Abcam, ab144269); or a pyruvate kinase M2 (PKM2) inhibitor, shikonin (5,8-dihydroxy-2-[(1R)-1-hydroxy-4-methyl-3-penten-1-yl]-1,4-naphthalenedione, 3 µM, [used in a previous study at 1 or 10 µM for B16 cells],^28^ Sigma-Aldrich, St. Louis, MO, USA, S7576). Citric acid cycle inhibition was induced by 5 µM UK5099 ((E)-2-Cyano-3-(1-phenyl-1H-indol-3-yl) acrylic acid, (E)-2-Cyano-3-(1-phenyl-1H-indol-3-yl)-2-propenoic acid, [used in a previous study at 10 µM for LNCaP cells],^29^ Funakoshi Co., Ltd., Tokyo, Japan, AG-CR1-3691-M0005), a mitochondrial pyruvate carrier inhibitor.

To measure relative ATP levels, HeLa cells were cultured for 24 hours in medium with or without lonidamine, heptelidic acid, and BCAAs, or for 48 hours in medium with or without UK5099 and BCAAs.

The 661W cells^30^ were kindly provided by Dr. Muayyad R. Al-Ubaidi (University of Houston, Houston, TX, USA). Glucose transporters (GLUT) inhibition was induced by WZB117 (Sigma; SML0621, an inhibitor of GLUT1, 3 and 4, used in a previous study at 0.7–60 µM for erythrocytes).^31^ The 661W cells were cultured in DMEM containing 4.5 g/L of glucose and 0.1% fetal bovine serum without amino acids and with or without 40 mM BCAAs for 2 hours, and then incubated with or without BCAAs and 100 µM WZB117 for 2 hours before measurement of relative ATP levels.

Relative ATP levels in cultured cells were measured by luciferase activity using an intracellular ATP assay kit (Toyo B-net, Tokyo, Japan) with a Nivo Multimode Microplate Reader (PerkinElmer, Inc., Waltham, MA, USA). After trypsinization, live cell numbers were measured with a TC20 cell counter (Bio-Rad, Hercules, CA, USA).

### Flowcytometry Analysis

To quantify the glucose uptake, the fluorescence of fluorescently labeled deoxy-glucose analog (2-NBDG; Wako) was quantified using flow cytometry. Tunicamycin (Nacalai Tesque, Kyoto, Japan) was added to induce ER stress, 2-deoxy-D-glucose (2-DG, Nacalai Tesque, hexokinase inhibitor) was added to inhibit glycolysis.

HeLa cells were cultured in DMEM containing 4.5 g/L glucose and 0.1% fetal bovine serum without amino acids for 24 hours, with or without 40 mM BCAAs, with or without 7 µg/mL tunicamycin, and with or without 75 mM 2-DG. The cells were then incubated in DMEM without glucose without amino acids containing 100 µM 2-NBDG for 15 minutes, with or without 40 mM BCAAs, with or without 7 µg/mL tunicamycin, and with or without 75 mM 2-DG before flow cytometry analysis.

HeLa cells and 661W cells were cultured in DMEM containing 4.5 g/L glucose and 0.1% fetal bovine serum without amino acids with or without BCAAs (0.04, 4.0, or 40 mM) for 24 hours, followed by culture with 100 µM 2-NBDG for 15 minutes before flow cytometry analysis.

HeLa cells and 661W cells were incubated in DMEM containing 4.5 g/L glucose and 0.1% fetal bovine serum without amino acids with or without 40 mM BCAAs for 3 hours. Next, the cells were incubated in DMEM containing 4.5 g/L glucose with or without 40 mM BCAAs, with or without WZB117 (250 or 100 µM for HeLa cells and 125 µM for 661W cells) for 1 hour before incubation with 100 µM 2-NBDG for 15 minutes.

The number of live cells and the fluorescence of 2-NBDG taken into the cells were quantified using FACS Calibur (BD Biosciences, Franklin Lakes, NJ, USA; Supplementary Fig. S2).

### Transfection of EGFP-GLUT1 or GLUT3 and Fluorescence Microscopy

Vector plasmids carrying enhanced green fluorescent protein (EGFP) fusion-GLUT1 or GLUT3 were prepared using the pEGFP-C2 vector (Clontech Laboratories, Mountain View, CA, USA; 6083-1). GLUT1 and GLUT3 cDNA was amplified from HeLa cells using the following primers: GLUT1, primer-F: CTCAAGCTTCGAATTCATGGAGCCCAGCAGCAA, and primer-R: TAGATCCGGTGGATCTCACACTTGGGAATCAGCC; GLUT3, primer-F: CTCAAGCTTCGAATTGATGGGGACACAGAAGGT, and primer-R: TAGATCCGGTGGATCTTAGACATTGGTGGTGGTC. The N-terminal of GLUT1 and GLUT3 was fused with EGFP. HeLa cells and 661W cells were transfected with the prepared vectors using Lipofectamine 3000 reagent (Thermo Fisher Scientific, Waltham, MA, USA). EGFP-GLUT1 or GLUT3 expression in HeLa or 661W cells was confirmed by Western blot analysis (Supplementary Fig. S3).

HeLa cells and 661W cells expressing EGFP-GLUT1 or GLUT3 were incubated in DMEM containing 4.5 g/L of glucose and 0.1% fetal bovine serum without amino acids with or without 80 mM BCAAs. Cells were imaged using a fluorescence microscope (BZ-9000, Keyence, Osaka, Japan) at 0, 60, 120, and 180 minutes after incubation. The fluorescence intensities of EGFP-GLUTs were quantified using BZ-II Analyzer software (Keyence). The ratio of fluorescence intensities of the plasma membrane to the cytosol was calculated.

### Statistical Analysis

Data are presented as the mean ± standard deviation. A Tukey honestly significant difference (HSD) test was used to compare parameters under multiple conditions in HeLa and 661W cells. The unpaired *t*-test was used to compare glucose uptake in HeLa cells, with or without BCAAs. Statistical significance was set at *P* < 0.05.

## Results

### BCAA Supplementation Raises ATP Levels in Cultured Cells During Glycolysis or Citric Acid Cycle Inhibition

**Figure 1.**
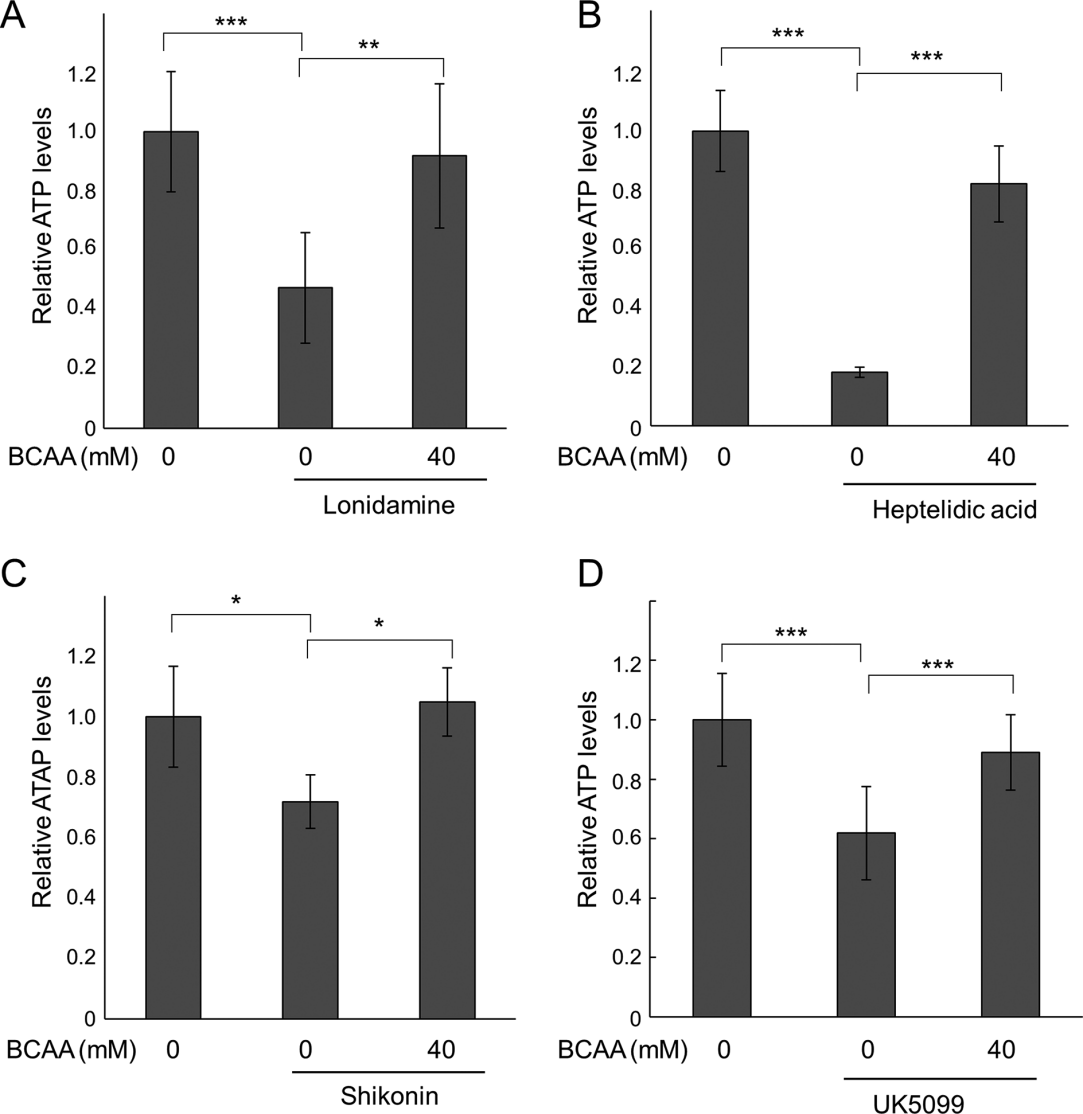
BCAAs recovered intracellular ATP decrease in cultured cells. HeLa cells were cultured in an amino acid-free medium containing 4.5 g/L of glucose with or without 40 mM Branched-chain amino acids (BCAAs), and with or without 300 µM lonidamine (glycolysis hexokinase inhibitor, **A**, *N* = 6), 0.6 µM heptelidic acid (glycolysis glyceraldehyde 3-phosphate dehydrogenase [GAPDH] inhibitor, **B**, *N* = 4), Shikonin (glycolysis pyruvate kinase M2 [PKM2] inhibitor, **C**, 3 µM, *N* = 4), or 5 µM UK5099 (citric acid cycle inhibitor, **D**, *N* = 7). After HeLa cells were cultured for 24 hours (**A**–**C**) or 48 hours (**D**), relative intracellular ATP levels were determined by luciferase activity. *P < 0.05, **P < 0.01, and ***P < 0.005, Tukey honestly significant difference (HSD). Bars represent standard deviation.

In HeLa cells, inhibition of glycolysis or citric acid cycle decreased intracellular ATP concentration (*P* = 0.002, *P* < 0.0001, *P* = 0.03, and *P* < 0.0001, lonidamine [glycolysis hexokinase inhibitor], heptelidic acid [glycolysis GAPDH inhibitor], Shikonin [glycolysis PKM2 inhibitor], and UK5099 [citric acid cycle mitochondrial pyruvate carrier inhibitor], respectively, Tukey HSD; Fig. 1). The addition of a formulation of BCAAs, L-isoleucine, L-leucine, and L-valine (1:2:1.2), recovered intracellular ATP levels during glycolysis inhibition (*P* = 0.006, *P* < 0.0001, and *P* = 0.013, lonidamine, heptelidic acid, and Shikonin, respectively, Tukey HSD; see Figs. 1A–C, Supplementary Fig. S1), and during citric acid cycle inhibition (*P* = 0.002, Tukey HSD; see Fig. 1D). BCAAs at ≥4 mM recovered the decreased intracellular ATP levels and live cell numbers during glycolysis inhibition induced by lonidamine (Supplementary Fig. S4). This BCAA concentration also increased ATP levels to prevent cell death under ER stress, as observed in our previous study,^14^ and was 10-fold higher than the human plasma concentration in the static state.^32^

Thus, BCAAs recovered the decrease in ATP levels induced by glycolysis inhibitors, which inhibit hexokinase, GAPDH, or PKM2, and by a citric acid cycle inhibitor, which inhibits mitochondrial pyruvate carrier activity. In our previous study, BCAAs also recovered ATP levels during mitochondrial respiratory chain inhibition.^14^

### BCAA Supplementation Promotes Glucose Uptake Thorough Glucose Transporter

**Figure 2.**
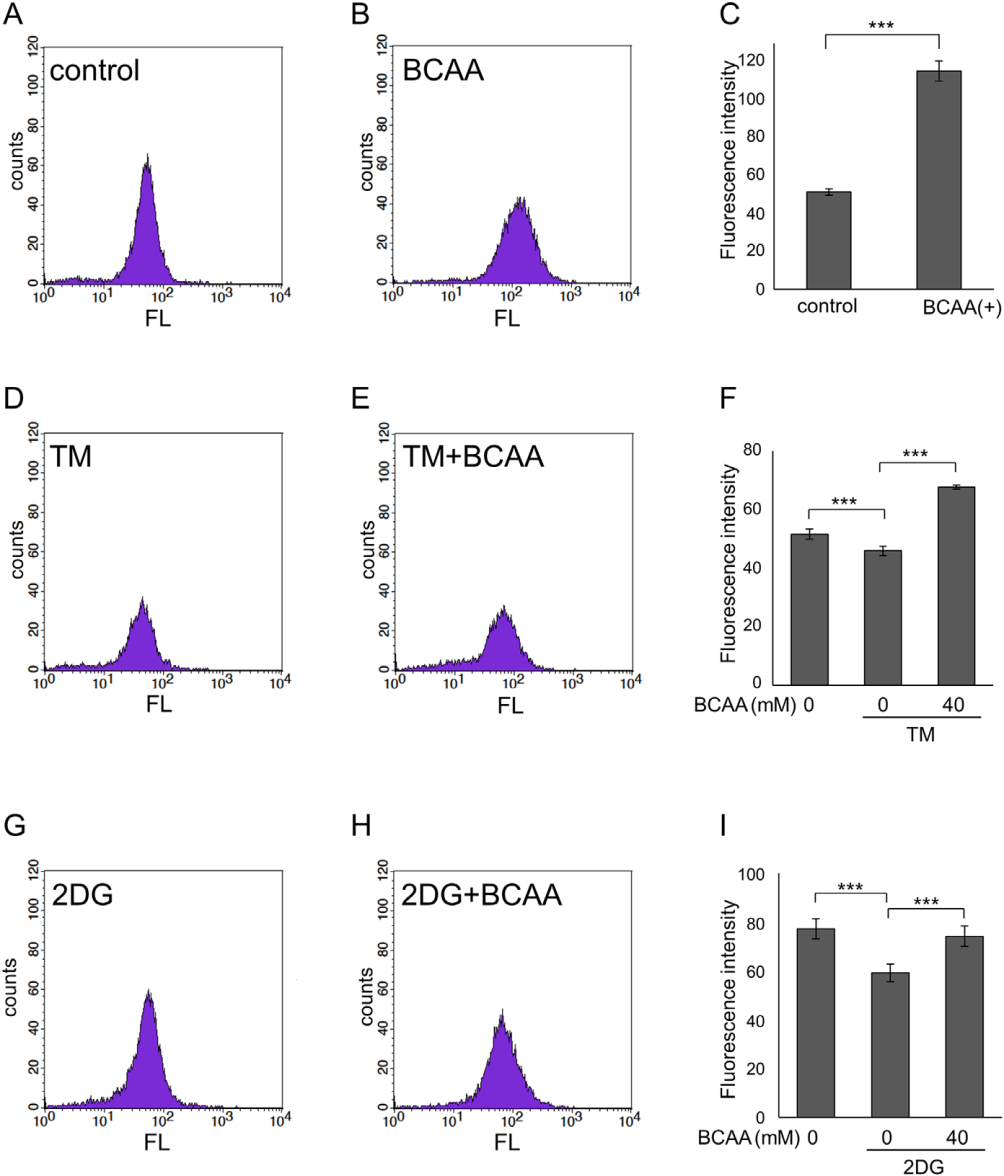
BCAAs promoted glucose uptake in HeLa cells. HeLa cells were cultured in an amino acid-free medium containing 4.5 g/L of glucose with or without 40 mM BCAAs (**A**–**I**) and with or without 7 µg/mL tunicamycin (TM; endoplasmic reticulum stress inducer) (**D**-**F**) or 75 mM 2-deoxy-D-glucose (2-DG, glycolysis hexokinase inhibitor) (**G**–**I**) for 24 hours before addition of fluorescently labeled deoxy-glucose analog (100 µM 2-NBDG). **A**, **B**, **D**, **E**, **G**, **H** Histograms showing distribution of cell counts for fluorescence intensities (FL) by flowcytometry analysis. **C**, **F**, **I** Geometric average of fluorescence intensities of live cells. **C** ***P < 0.0001, unpaired *t*-test, **F**, **I** ***P < 0.005, Tukey HSD, *N* = 4. Bars represent the standard deviation.

Next, we investigated the effect of BCAAs on glucose uptake. Glucose uptake quantified by measuring the fluorescence of 2-NBDG using flow cytometry was significantly increased by addition of BCAAs in HeLa cells (*P* < 0.0001, unpaired *t*-test; Figs. 2A–C). BCAAs at ≥4 mM increased glucose uptake (Supplementary Fig. S5A). Glucose uptake was significantly decreased when ER stress was induced by tunicamycin (*P* = 0.001, Tukey HSD), and glucose uptake was recovered by BCAA supplementation (*P* < 0.0001, Tukey HSD; see Figs. 2D–F). Glucose uptake was also significantly decreased when glycolysis was inhibited by 2-DG (*P* < 0.0001, Tukey HSD), and glucose uptake was recovered by BCAA supplementation (*P* = 0.001, Tukey HSD; see Figs. 2G–I).

**Figure 3.**
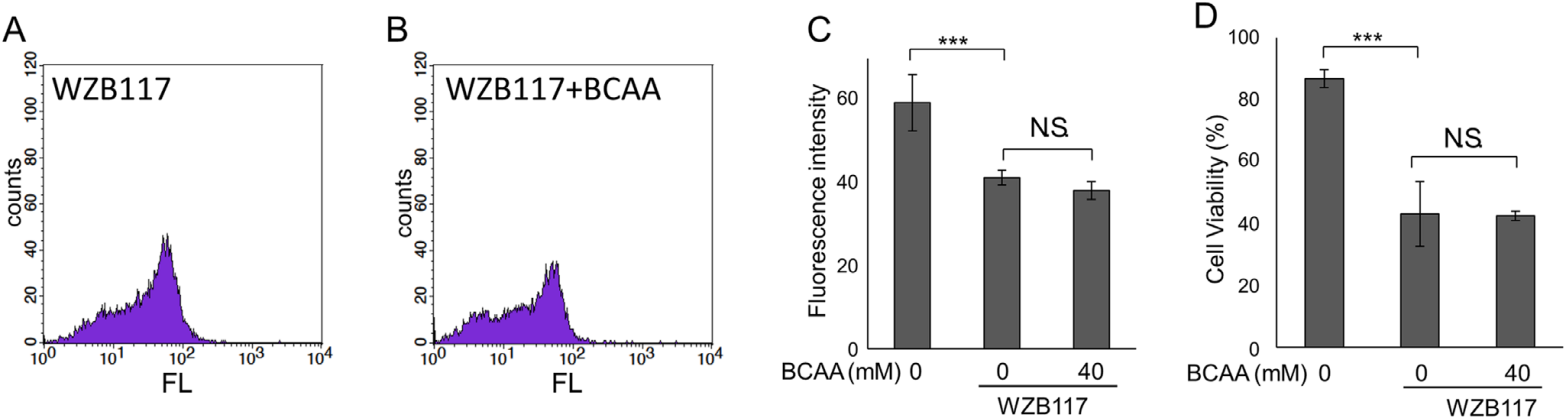
Inhibition of glucose transporters prevented BCAAs from promoting glucose uptake. HeLa cells were cultured in an amino acid-free medium containing 4.5 g/L of glucose with or without 40 mM BCAAs for 3 hours and then incubated with or without 250 µM WZB117 (inhibitor of glucose transporter [GLUT] 1, 3, and 4) for 1 hour before addition of fluorescently labeled deoxy-glucose analog (100 µM 2-NBDG). **A**–**C** Glucose uptake with or without BCAAs and with or without WZB117 in HeLa cells. **A**, **B** Histograms showing distributions of cell counts for fluorescence intensities (FL) by flowcytometry analysis. **C** Geometric average of fluorescence intensities of live cells. **D** Cell viability with or without BCAAs and with or without WZB117 in HeLa cells. **C**, **D** ***P < 0.005, N.S. no significant difference, Tukey HSD, *N* = 4 each. Bars represent standard deviation.

Glucose uptake by cells is mediated by glucose transporters (GLUTs).^33^ GLUT1, GLUT3, and GLUT4 have been reported to be expressed in retinal cells.^34^^–^^38^ For this reason, we studied the effect of BCAA supplementation on GLUT inhibition. The addition of WZB117, an inhibitor of GLUT1, 3, and 4, resulted in suppression of glucose uptake in HeLa cells (*P* < 0.001, Tukey HSD), and this was not recovered by BCAAs (*P* = 0.58, Tukey HSD; Figs. 3A–C, Supplementary Fig. S6A). Under these conditions, cell viability decreased (*P* < 0.0001, Tukey HSD), and did not recover by addition of BCAAs (*P* = 0.99, Tukey HSD; see Fig. 3D, Supplementary Fig. S6B).

**Figure 4.**
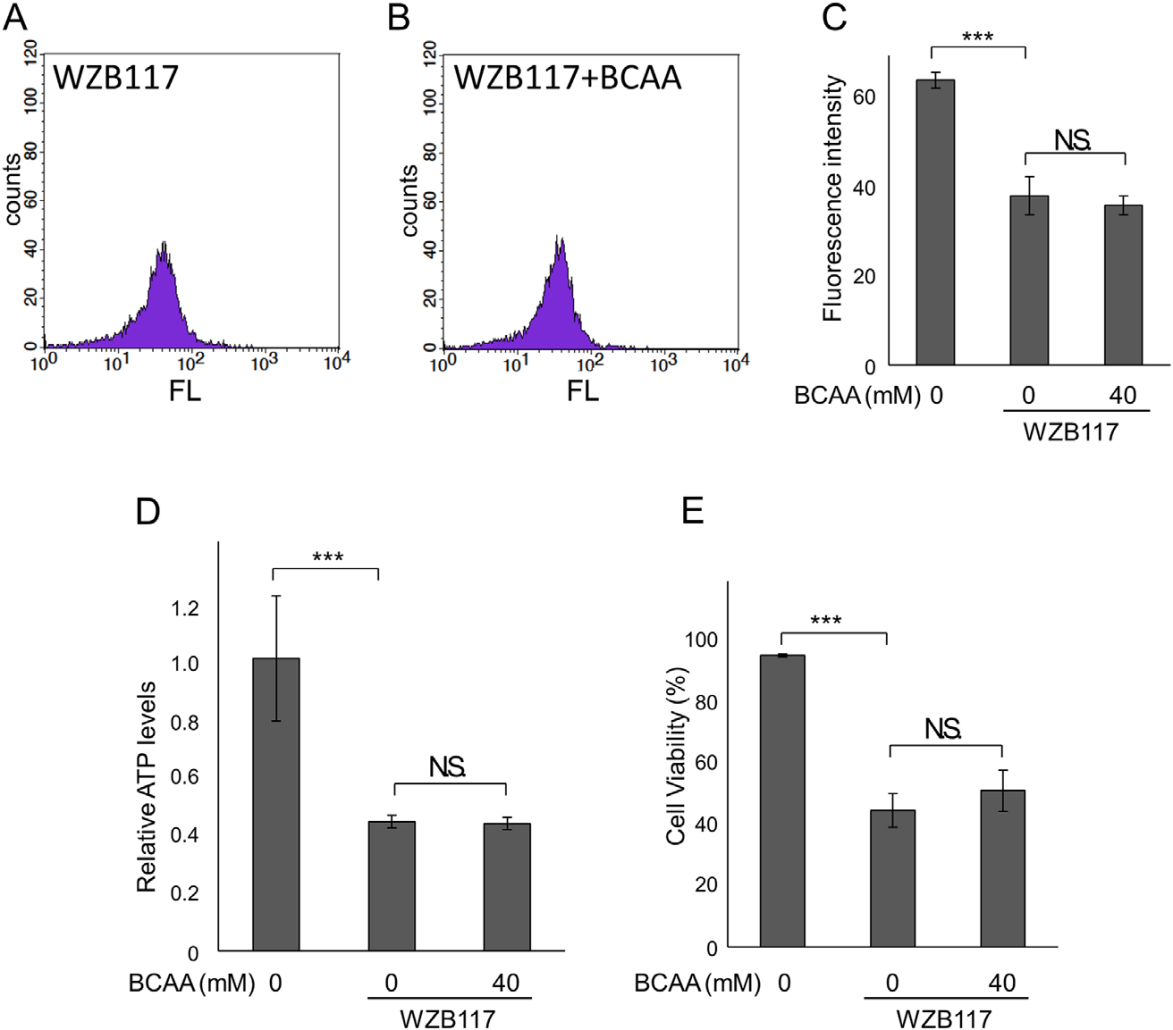
Inhibition of glucose transporters prevented BCAAs from promoting glucose uptake in photoreceptor-derived 661W cells. Retinal photoreceptor-derived cells, 661W cells, were cultured in an amino acid-free medium containing 4.5 g/L of glucose with or without 40 mM BCAAs, and then incubated with or without WZB117. **A**–**C** Glucose uptake quantified after incubation with 100 µM 2-NBDG after incubation with or without BCAAs for 3 hours and then with or without WZB117 (125 µM) for 1 hour. **A**, **B** Histograms showing distributions of cell counts for fluorescence intensities (FL) by flowcytometry analysis. **C** Geometric average of fluorescence intensities of live cells. **D** Relative ATP level in live cells after incubation with or without BCAAs for 2 hours and then with or without 100 µM WZB117 for 2 hours. **E** Cell viability with or without BCAAs and with or without 125 µM WZB117. **C**–**E** ***P < 0.005, N.S.: no significant difference, Tukey HSD, **C**, **E** *N* = 4 and **D** *N* = 3. Bars represent the standard deviation.

Next, we evaluated glucose uptake in retinal photoreceptor-derived cells, 661W.^30^ BCAAs also increased glucose uptake by 661W cells (see Supplementary Fig. S5B). Glucose uptake in 661W cells was decreased by the addition of WZB117 (*P* < 0.0001, Tukey HSD), and was not recovered by BCCAs supplementation (*P* = 0.57, Tukey HSD; Figs. 4A–C). Under these conditions, the intracellular ATP concentration also decreased (*P* = 0.004, Tukey HSD), and did not recover by addition of BCAAs (*P* = 0.997, Tukey HSD; see Fig. 4D). Similarly, cell viability decreased (*P* < 0.0001, Tukey HSD), and did not recover by the addition of BCAAs (*P* = 0.22, Tukey HSD; see Fig. 4E).

These results show that BCAA supplementation promotes glucose uptake through GLUTs and recovers intracellular ATP levels.

### BCAA Supplementation Promotes Translocation of Glucose Transporter to Cellular Surface Membrane

Next, we studied the effect of BCAAs on the expression of GLUTs. First, HeLa cells were cultured with or without BCAAs. Western blotting analysis of the whole cells showed no apparent differences in the expression of GLUT1 and GLUT3 among the various conditions (Supplementary Fig. S7A–C). Then, we evaluated GLUT1 expression on the plasma membrane under stress conditions. HeLa cells were cultured with or without BCAAs and with or without tunicamycin, and the plasma membrane fraction was extracted. The amount of GLUT1 protein in the plasma membrane was less in tunicamycin-treated cells (*P* = 0.02, Tukey HSD) in comparison with non-treated cells (see Supplementary Figs. S7D–E).

**Figure 5.**
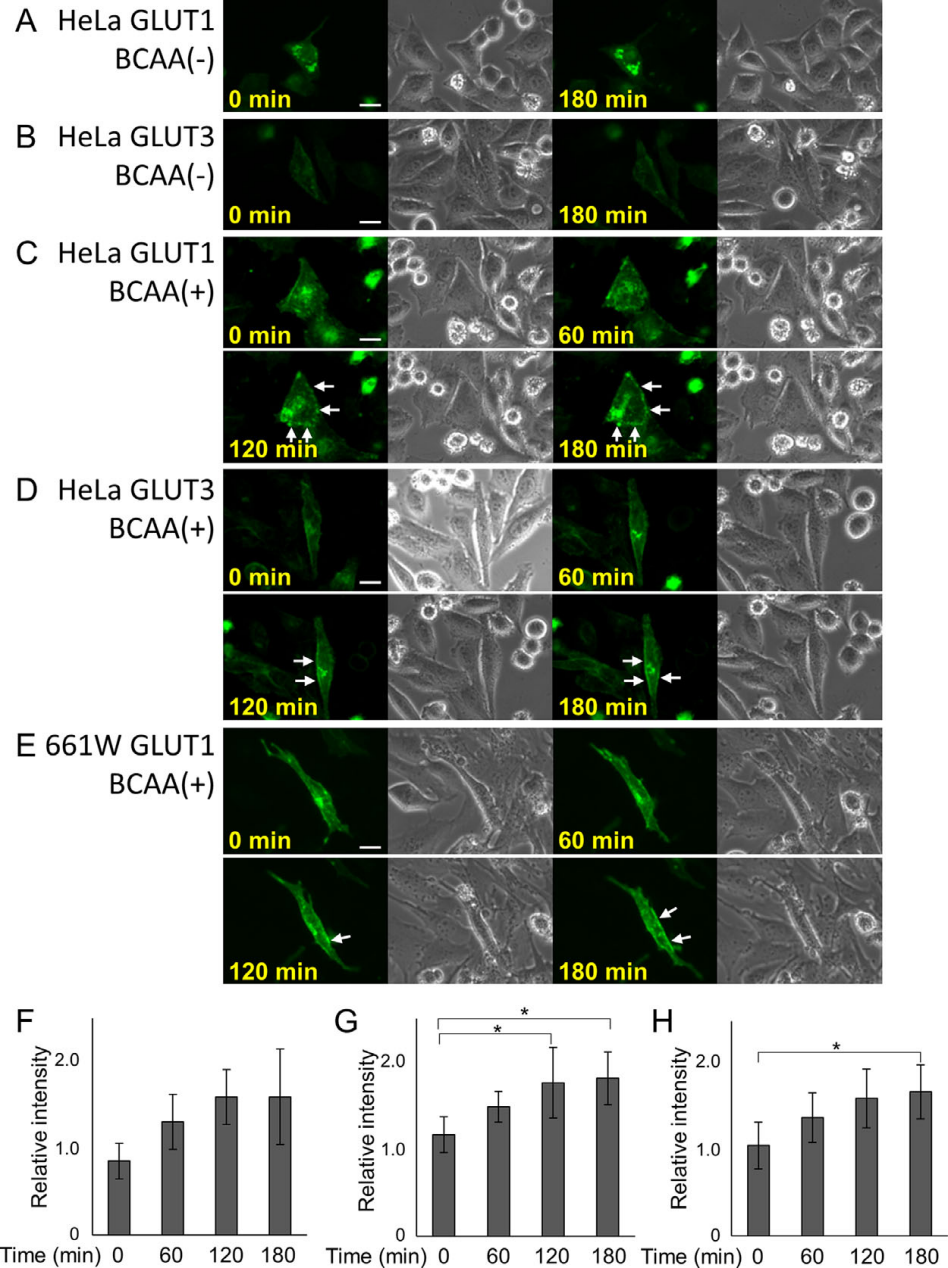
BCAAs promoted glucose transporters (GLUT) translocation to the plasma membrane. HeLa cells and 661W cells expressing enhanced green fluorescent protein (EGFP)-GLUT1 or GLUT3 were incubated in an amino acid-free medium containing 4.5 g/L of glucose with or without BCAAs and imaged under fluorescence microscope at 0, 60, 120, and 180 minutes after incubation. **A**, **B** HeLa cells expressing EGFP-GLUT1 (**A**) or EGFP-GLUT3 (**B**) were incubated without BCAAs. **C**, **D**, **F**, **G** HeLa cells expressing EGFP-GLUT1 (**C**, **F**) or EGFP-GLUT3 (**D**, **G**) were incubated with 80 mM BCAAs. **E**, **H** 661W cells expressing EGFP-GLUT1 were incubated with 80 mM BCAAs. White arrows indicate EGFP-fusion GLUTs translocated to the plasma membrane (**C**–**E**). Scale: 20 µm. **F**, **G**, **H** Fluorescence intensities of EGFP-GLUT1 (**F**, **H**) or GLUT3 (**G**) were quantified in HeLa cells (**F**, **G**) or 661W cells (**H**) at 0, 60, 120, and 180 minutes after incubation. Fluorescence intensities are shown as the ratio of that at the plasma membrane to that in the cytosol. *P < 0.05, Tukey HSD, *N* = 5 each.

To confirm whether the translocation of GLUTs to the plasma membrane is promoted by BCAAs, we transfected EGFP-GLUT1 or GLUT3 in HeLa or 661W cells, to observe the intracellular behavior of GLUT1 and GLUT3. Fluorescence microscopy revealed that EGFP-GLUT proteins remained in the cytoplasm in cells cultured without BCAA supplementation, and these proteins were translocated to the plasma membrane over time, in the cells supplemented with BCAAs (Fig. 5, *P* = 0.052, *P* = 0.026, and *P* = 0.046, 0 vs. 180 minutes, for GLUT1 in HeLa cells, GLUT3 in HeLa cells and GLUT1 in 661W cells, respectively).

## Discussion

In the current study, we showed that BCAAs prevented the decrease in intracellular ATP even when glycolysis or the citric acid cycle was inhibited. Moreover, we clearly showed that BCAAs enhanced the translocation of GLUTs to the plasma membrane, and promoted glucose uptake.

We previously showed that BCAAs prevented the decrease in intracellular ATP and antagonized cell death during mitochondrial respiratory chain inhibition stress or ER stress.^14^ In the current study, we showed that BCAAs prevented decreases in intracellular ATP even when glycolysis or citric acid cycle was inhibited by a hexokinase inhibitor (lonidamine), a GAPDH inhibitor (heptelidic acid), a PKM2 inhibitor (Shikonin), or mitochondrial pyruvate carrier inhibitor (UK5099) as well (see Fig. 1). Fluorescence imaging of glucose uptake using a fluorescently labeled deoxy-glucose analog showed that BCAAs promoted glucose uptake (see Fig. 2). However, when hexokinase was inhibited by 2-DG, intracellular ATP levels further decreased following addition of BCAAs in our previous study,^14^ whereas glucose uptake was enhanced by BCAAs in the presence of 2-DG (see Figs. 2G–I). This result is reasonable because enhanced uptake of 2-DG by BCAAs further strengthened the feedback inhibition of hexokinase by 2-DG-6 phosphate. In contrast, BCAAs did not antagonize the decrease in ATP nor cell death when GLUTs were inhibited (see Figs. 3, 4), likely because GLUTs were the target of ATP enhancement by BCAAs.

Furthermore, in the current study, fluorescence imaging of EGFP-GLUTs (GLUT1 or GLUT3) apparently showed that BCAAs enhanced the translocation of GLUTs to the plasma membrane (see Fig. 5). Thus, BCAAs enhanced ATP production by promoting glucose uptake through promotion of GLUTs translocation to the plasma membrane, thereby preventing cell death.

The translocation of GLUTs to the plasma membrane has been shown to be induced by a variety of stimuli, including insulin,^39^^,^^40^ platelet-derived growth factor,^41^ and epidermal growth factor.^42^ In addition, BCAAs have been suggested to promote GLUT translocation to the plasma membrane, and consequently promote glucose uptake in skeletal muscle.^43^^,^^44^ Paradoxically, BCAAs have been reported to increase insulin resistance.^45^^,^^46^ Thus, the influence of BCAAs on glucose uptake or GLUTs is not fully understood.^17^ In the current study, we directly observed the intracellular behavior of GLUTs using transfected EGFP-GLUT1 or GLUT3. Fluorescent microscopy clearly revealed that BCAAs promoted the translocation of GLUTs to the plasma membrane in HeLa cells and in the photoreceptor-derived neuronal cells, 661W, consequently promoting glucose uptake, enhancing ATP production, and attenuating cell death.

We have previously shown that BCAAs attenuated photoreceptor degeneration in rodent models of retinal degeneration, and attenuated retinal ganglion cell death in a rodent model of glaucoma.^14^ Although high plasma concentrations of BCAAs are associated with a high incidence of cardiovascular disease, BCAA concentrations are not associated with cardiovascular diseases when HbA1c and insulin resistance were adjusted.^47^ Moreover, leucine has been reported to attenuate cardiac damage, and improve cardiac function and survival after acute myocardial infarction.^48^ In contrast, energy depletion has been associated with neuronal cell death, including in retinal neurons.^49^^–^^52^ Considering that BCAAs antagonize cell death in the heart and the retina, both of which are organs that require a large amount of energy,^53^^–^^56^ BCAAs might have the potential to protect cells against cell death during high energy requirement conditions.

This study has several limitations. First, the influence of each BCAA on glucose uptake was not investigated separately. Leucine and isoleucine have been reported to promote glucose uptake in skeletal muscle,^20^ and leucine is known to activate mammalian target of rapamycin (mTOR).^57^ Excess leucine without other amino acids has been reported to have growth-inhibitory effects.^58^ Because we aimed to clinically apply BCAAs for retinal neurodegenerative diseases, we studied the mechanisms of glucose uptake by formulation of BCAAs, leucine, isoleucine, and valine, which have already been used clinically in patients with liver cirrhosis.^24^ Second, the possible effect of BCAAs on insulin resistance was not studied in the current study. It has been suggested that leucine and isoleucine induce the translocation of GLUT1 and GLUT4, which is insulin-sensitive GLUT, to the plasma membrane.^43^ In the current study, we clearly showed that BCAAs promote the translocation of GLUTs to the plasma membrane, and consequently promote glucose uptake in cultured cells. Whereas insulin-sensitive GLUT4, which is mainly expressed in adipose tissue, skeletal muscle, and cardiomyocytes,^33^ has been reported to be expressed in retinal neuronal cells,^38^ GLUT1 has been reported to play the most important role in glucose uptake by cone photoreceptors.^59^ On the contrary, stimulation of insulin/mTOR pathways has been reported to attenuate cone photoreceptor cell death.^51^^,^^59^ The effect of insulin on glucose uptake in the presence of BCAAs requires further study.

In conclusion, BCAAs promote the translocation of GLUTs to the plasma membranes, and consequently increase intracellular ATP concentration by enhancing glucose uptake into the cells. BCAAs have the potential to be a novel therapeutic strategy for retinal neurodegenerative diseases by preventing decrease in intracellular ATP levels.

## Supplementary Material

Supplement 1

## References

[1] Bennett J, Ashtari M, Wellman J, et al. AAV2 gene therapy readministration in three adults with congenital blindness. *Sci Transl Med**.* 2012; 4: 120ra115. 10.1126/scitranslmed.3002865 PMC4169122 22323828

[2] Botto C, Rucli M, Tekinsoy MD, Pulman J, Sahel JA, Dalkara D. Early and late stage gene therapy interventions for inherited retinal degenerations. *Prog Retin Eye Res**.* 2022; 86: 100975. 34058340 10.1016/j.preteyeres.2021.100975

[3] Mannino G, Russo C, Longo A, et al. Potential therapeutic applications of mesenchymal stem cells for the treatment of eye diseases. *World J Stem Cells**.* 2021; 13: 632–644. 34249232 10.4252/wjsc.v13.i6.632 PMC8246249

[4] Birch DG, Bennett LD, Duncan JL, Weleber RG, Pennesi ME. Long-term Follow-up of Patients With Retinitis Pigmentosa Receiving Intraocular Ciliary Neurotrophic Factor Implants. *Am J Ophthalmol**.* 2016; 170: 10–14. 27457255 10.1016/j.ajo.2016.07.013 PMC5056139

[5] Sahel JA, Boulanger-Scemama E, Pagot C, et al. Partial recovery of visual function in a blind patient after optogenetic therapy. *Nat Med**.* 2021; 27: 1223–1229. 34031601 10.1038/s41591-021-01351-4

[6] Sacchetti M, Mantelli F, Merlo D, Lambiase A. Systematic Review of Randomized Clinical Trials on Safety and Efficacy of Pharmacological and Nonpharmacological Treatments for Retinitis Pigmentosa. *J Ophthalmol**.* 2015; 2015: 737053. 26339504 10.1155/2015/737053 PMC4539114

[7] Collaborative Normal-Tension Glaucoma Study Group. The effectiveness of intraocular pressure reduction in the treatment of normal-tension glaucoma. Collaborative Normal-Tension Glaucoma Study Group. *Am J Ophthalmol**.* 1998; 126: 498–505. 9780094 10.1016/s0002-9394(98)00272-4

[8] Nakano N, Ikeda HO, Hasegawa T, et al. Neuroprotective effects of VCP modulators in mouse models of glaucoma. *Heliyon**.* 2016; 2: e00096. 27441270 10.1016/j.heliyon.2016.e00096 PMC4946081

[9] Ikeda HO, Sasaoka N, Koike M, et al. Novel VCP modulators mitigate major pathologies of rd10, a mouse model of retinitis pigmentosa. *Sci Rep*. 2014; 4: 5970. 25096051 10.1038/srep05970 PMC4122966

[10] Hasegawa T, Muraoka Y, Ikeda HO, et al. Neuoroprotective efficacies by KUS121, a VCP modulator, on animal models of retinal degeneration. *Sci Rep**.* 2016; 6: 31184. 27503804 10.1038/srep31184 PMC4977562

[11] Muraoka Y, Iida Y, Ikeda HO, et al. KUS121, an ATP regulator, mitigates chorioretinal pathologies in animal models of age-related macular degeneration. *Heliyon**.* 2018; 4: e00624. 29872758 10.1016/j.heliyon.2018.e00624 PMC5986307

[12] Hata M, Ikeda HO, Kikkawa C, et al. KUS121, a VCP modulator, attenuates ischemic retinal cell death via suppressing endoplasmic reticulum stress. *Sci Rep**.* 2017; 7: 44873. 28317920 10.1038/srep44873 PMC5357950

[13] Ikeda HO, Muraoka Y, Hata M, et al. Safety and effectiveness of a novel neuroprotectant, KUS121, in patients with non-arteritic central retinal artery occlusion: An open-label, non-randomized, first-in-humans, phase 1/2 trial. *PLoS One**.* 2020; 15: e0229068. 32053676 10.1371/journal.pone.0229068 PMC7018138

[14] Hasegawa T, Ikeda HO, Iwai S, et al. Branched chain amino acids attenuate major pathologies in mouse models of retinal degeneration and glaucoma. *Heliyon**.* 2018; 4: e00544. 29560458 10.1016/j.heliyon.2018.e00544 PMC5857634

[15] Hasegawa T, Ikeda HO. Adenosine triphosphate maintenance by branched chain amino acids as a novel neuroprotective strategy for retinal neurodegenerative diseases. *Neural Regen Res**.* 2019; 14: 82–84. 30531080 10.4103/1673-5374.244788 PMC6263008

[16] Mikalayeva V, Pankevičiūtė M, Žvikas V, Skeberdis VA, Bordel S. Contribution of branched chain amino acids to energy production and mevalonate synthesis in cancer cells. *Biochem Biophys Res Commun*. 2021; 585: 61–67. 34794035 10.1016/j.bbrc.2021.11.034

[17] Yoneshiro T, Wang Q, Tajima K, et al. BCAA catabolism in brown fat controls energy homeostasis through SLC25A44. *Nature**.* 2019; 572: 614–619. 31435015 10.1038/s41586-019-1503-x PMC6715529

[18] Kamei Y, Hatazawa Y, Uchitomi R, Yoshimura R, Miura S. Regulation of Skeletal Muscle Function by Amino Acids. *Nutrients**.* 2020; 12: 261. 10.3390/nu12010261 PMC7019684 31963899

[19] Matsumura T, Morinaga Y, Fujitani S, Takehana K, Nishitani S, Sonaka I. Oral administration of branched-chain amino acids activates the mTOR signal in cirrhotic rat liver. *Hepatol Res**.* 2005; 33: 27–32. 16169275 10.1016/j.hepres.2005.07.001

[20] Doi M, Yamaoka I, Nakayama M, Mochizuki S, Sugahara K, Yoshizawa F. Isoleucine, a blood glucose-lowering amino acid, increases glucose uptake in rat skeletal muscle in the absence of increases in AMP-activated protein kinase activity. *J Nutr**.* 2005; 135: 2103–2108. 16140883 10.1093/jn/135.9.2103

[21] Nishitani S, Matsumura T, Fujitani S, Sonaka I, Miura Y, Yagasaki K. Leucine promotes glucose uptake in skeletal muscles of rats. *Biochem Biophys Res Commun**.* 2002; 299: 693–696. 12470633 10.1016/s0006-291x(02)02717-1

[22] Nie C, He T, Zhang W, Zhang G, Ma X. Branched Chain Amino Acids: Beyond Nutrition Metabolism. *Int J Mol Sci*. 2018; 19: 954. 10.3390/ijms19040954 PMC5979320 29570613

[23] Muto Y, Sato S, Watanabe A, et al. Effects of oral branched-chain amino acid granules on event-free survival in patients with liver cirrhosis. *Clin Gastroenterol Hepatol**.* 2005; 3: 705–713. 16206505 10.1016/s1542-3565(05)00017-0

[24] Plauth M, Cabre E, Riggio O, et al. ESPEN Guidelines on Enteral Nutrition: Liver disease. *Clin Nutr**.* 2006; 25: 285–294. 16707194 10.1016/j.clnu.2006.01.018

[25] Kawaguchi T, Shiraishi K, Ito T, et al. Branched-chain amino acids prevent hepatocarcinogenesis and prolong survival of patients with cirrhosis. *Clin Gastroenterol Hepatol**.* 2014; 12: 1012–1018.e1011. 24036055 10.1016/j.cgh.2013.08.050

[26] Floridi A, Paggi MG, D'Atri S, et al. Effect of lonidamine on the energy metabolism of Ehrlich ascites tumor cells. *Cancer Res**.* 1981; 41: 4661–4666. 7306982

[27] Kumagai S, Narasaki R, Hasumi K. Glucose-dependent active ATP depletion by koningic acid kills high-glycolytic cells. *Biochem Biophys Res Commun**.* 2008; 365: 362–368. 17997978 10.1016/j.bbrc.2007.10.199

[28] Zhao X, Zhu Y, Hu J, et al. Shikonin Inhibits Tumor Growth in Mice by Suppressing Pyruvate Kinase M2-mediated Aerobic Glycolysis. *Sci Rep*. 2018; 8: 14517. 30266938 10.1038/s41598-018-31615-y PMC6162216

[29] Zhong Y, Li X, Yu D, et al. Application of mitochondrial pyruvate carrier blocker UK5099 creates metabolic reprogram and greater stem-like properties in LnCap prostate cancer cells in vitro. *Oncotarget*. 2015; 6: 37758–37769. 26413751 10.18632/oncotarget.5386 PMC4741963

[30] Al-Ubaidi MR, Matsumoto H, Kurono S, Singh A. Proteomics profiling of the cone photoreceptor cell line, 661W. *Adv Exp Med Biol**.* 2008; 613: 301–311. 18188958 10.1007/978-0-387-74904-4_35

[31] Ojelabi OA, Lloyd KP, Simon AH, De Zutter JK, Carruthers A. WZB117 (2-Fluoro-6-(m-hydroxybenzoyloxy) Phenyl m-Hydroxybenzoate) Inhibits GLUT1-mediated Sugar Transport by Binding Reversibly at the Exofacial Sugar Binding Site. *J Biol Chem**.* 2016; 291: 26762–26772. 27836974 10.1074/jbc.M116.759175 PMC5207184

[32] Borgenvik M, Apró W, Blomstrand E. Intake of branched-chain amino acids influences the levels of MAFbx mRNA and MuRF-1 total protein in resting and exercising human muscle. *Am J Physiol Endocrinol Metab**.* 2012; 302: E510–521. 22127230 10.1152/ajpendo.00353.2011

[33] Mueckler M, Thorens B. The SLC2 (GLUT) family of membrane transporters. *Mol Aspects Med**.* 2013; 34: 121–138. 23506862 10.1016/j.mam.2012.07.001 PMC4104978

[34] Swarup A, Samuels IS, Bell BA, et al. Modulating GLUT1 expression in retinal pigment epithelium decreases glucose levels in the retina: impact on photoreceptors and Muller glial cells. *Am J Physiol Cell Physiol**.* 2019; 316: C121–C133. 30462537 10.1152/ajpcell.00410.2018 PMC6383144

[35] Hsu SC, Molday RS. Glycolytic enzymes and a GLUT-1 glucose transporter in the outer segments of rod and cone photoreceptor cells. *J Biol Chem**.* 1991; 266: 21745–21752. 1939198

[36] Kumagai AK, Glasgow BJ, Pardridge WM. GLUT1 glucose transporter expression in the diabetic and nondiabetic human eye. *Invest Ophthalmol Vis Sci**.* 1994; 35: 2887–2894. 8188484

[37] Badr GA, Zhang JZ, Tang J, Kern TS, Ismail-Beigi F. Glut1 and glut3 expression, but not capillary density, is increased by cobalt chloride in rat cerebrum and retina. *Brain Res Mol Brain Res**.* 1999; 64: 24–33. 9889305 10.1016/s0169-328x(98)00301-5

[38] Sánchez-Chávez G, Peña-Rangel MT, Riesgo-Escovar JR, Martínez-Martínez A, Salceda R. Insulin stimulated-glucose transporter Glut 4 is expressed in the retina. *PLoS One*. 2012; 7: e52959. 23285235 10.1371/journal.pone.0052959 PMC3528717

[39] Kolter T, Uphues I, Wichelhaus A, Reinauer H, Eckel J. Contraction-induced translocation of the glucose transporter Glut4 in isolated ventricular cardiomyocytes. *Biochem Biophys Res Commun**.* 1992; 189: 1207–1214. 1472028 10.1016/0006-291x(92)92333-s

[40] Kanai F, Ito K, Todaka M, et al. Insulin-stimulated GLUT4 translocation is relevant to the phosphorylation of IRS-1 and the activity of PI3-kinase. *Biochem Biophys Res Commun*. 1993; 195: 762–768. 8396927 10.1006/bbrc.1993.2111

[41] Kamohara S, Hayashi H, Todaka M, et al. Platelet-derived growth factor triggers translocation of the insulin-regulatable glucose transporter (type 4) predominantly through phosphatidylinositol 3-kinase binding sites on the receptor. *Proc Natl Acad Sci USA*. 1995; 92: 1077–1081. 7862637 10.1073/pnas.92.4.1077 PMC42640

[42] Ishii K, Kamohara S, Hayashi H, et al. Epidermal growth factor triggers the translocation of insulin-responsive glucose transporter (GLUT4). *Biochem Biophys Res Commun**.* 1994; 205: 857–863. 7999123 10.1006/bbrc.1994.2743

[43] Nishitani S, Takehana K, Fujitani S, Sonaka I. Branched-chain amino acids improve glucose metabolism in rats with liver cirrhosis. *Am J Physiol Gastrointest Liver Physiol**.* 2005; 288: G1292–1300. 15591158 10.1152/ajpgi.00510.2003

[44] Zhang S, Yang Q, Ren M, et al. Effects of isoleucine on glucose uptake through the enhancement of muscular membrane concentrations of GLUT1 and GLUT4 and intestinal membrane concentrations of Na+/glucose co-transporter 1 (SGLT-1) and GLUT2. *Br J Nutr*. 2016; 116: 593–602. 27464458 10.1017/S0007114516002439

[45] Zhao X, Han Q, Liu Y, Sun C, Gang X, Wang G. The Relationship between Branched-Chain Amino Acid Related Metabolomic Signature and Insulin Resistance: A Systematic Review. *J Diabetes Res**.* 2016; 2016: 2794591. 27642608 10.1155/2016/2794591 PMC5014958

[46] Lynch CJ, Adams SH. Branched-chain amino acids in metabolic signalling and insulin resistance. *Nat Rev Endocrinol*. 2014; 10: 723–736. 25287287 10.1038/nrendo.2014.171 PMC4424797

[47] Tobias DK, Lawler PR, Harada PH, et al. Circulating Branched-Chain Amino Acids and Incident Cardiovascular Disease in a Prospective Cohort of US Women. *Circ Genom Precis Med**.* 2018; 11: e002157. 29572205 10.1161/CIRCGEN.118.002157 PMC5880282

[48] Witham WG, Yester KA, McGaffin KR. A high leucine diet mitigates cardiac injury and improves survival after acute myocardial infarction. *Metabolism*. 2013; 62: 290–302. 22935555 10.1016/j.metabol.2012.07.023

[49] Lin MT, Beal MF. Mitochondrial dysfunction and oxidative stress in neurodegenerative diseases. *Nature**.* 2006; 443: 787–795. 17051205 10.1038/nature05292

[50] Liang WS, Reiman EM, Valla J, et al. Alzheimer's disease is associated with reduced expression of energy metabolism genes in posterior cingulate neurons. *Proc Natl Acad Sci USA**.* 2008; 105: 4441–4446. 18332434 10.1073/pnas.0709259105 PMC2393743

[51] Punzo C, Kornacker K, Cepko CL. Stimulation of the insulin/mTOR pathway delays cone death in a mouse model of retinitis pigmentosa. *Nat Neurosci**.* 2009; 12: 44–52. 19060896 10.1038/nn.2234 PMC3339764

[52] Umino Y, Everhart D, Solessio E, et al. Hypoglycemia leads to age-related loss of vision. *Proc Natl Acad Sci USA**.* 2006; 103: 19541–19545. 17159157 10.1073/pnas.0604478104 PMC1697832

[53] Li Q, Zhou LY, Gao GF, Jiao JQ, Li PF. Mitochondrial network in the heart. *Protein Cell*. 2012; 3: 410–418. 22752872 10.1007/s13238-012-2921-9 PMC4875487

[54] Futterman S, Kinoshita JH. Metabolism of the retina. I. Respiration of cattle retina. *J Biol Chem**.* 1959; 234: 723–726. 13654250

[55] Winkler BS. Glycolytic and oxidative metabolism in relation to retinal function. *J Gen Physiol**.* 1981; 77: 667–692. 6267165 10.1085/jgp.77.6.667 PMC2215447

[56] Chertov AO, Holzhausen L, Kuok IT, et al. Roles of glucose in photoreceptor survival. *J Biol Chem**.* 2011; 286: 34700–34711. 21840997 10.1074/jbc.M111.279752 PMC3186402

[57] Bhaskar PT, Hay N. The two TORCs and Akt. *Dev Cell**.* 2007; 12: 487–502. 17419990 10.1016/j.devcel.2007.03.020

[58] Harper AE, Miller RH, Block KP. Branched-chain amino acid metabolism. *Annu Rev Nutr**.* 1984; 4: 409–454. 6380539 10.1146/annurev.nu.04.070184.002205

[59] Conart JB, Blot G, Augustin S, et al. Insulin inhibits inflammation-induced cone death in retinal detachment. *J Neuroinflammation*. 2020; 17: 358. 33243251 10.1186/s12974-020-02039-1 PMC7694924

