# Supplementary material for: Branched Chain Amino Acids Promote ATP Production Via Translocation of Glucose Transporters
Source: Invest Ophthalmol Vis Sci. 2022 Aug 5;63(9):7. doi: 10.1167/iovs.63.9.7 (PMC9363681; doi:10.1167/iovs.63.9.7)
Supplement: Supplement 1 [file iovs-63-9-7_s001.pdf]

Supplementary information

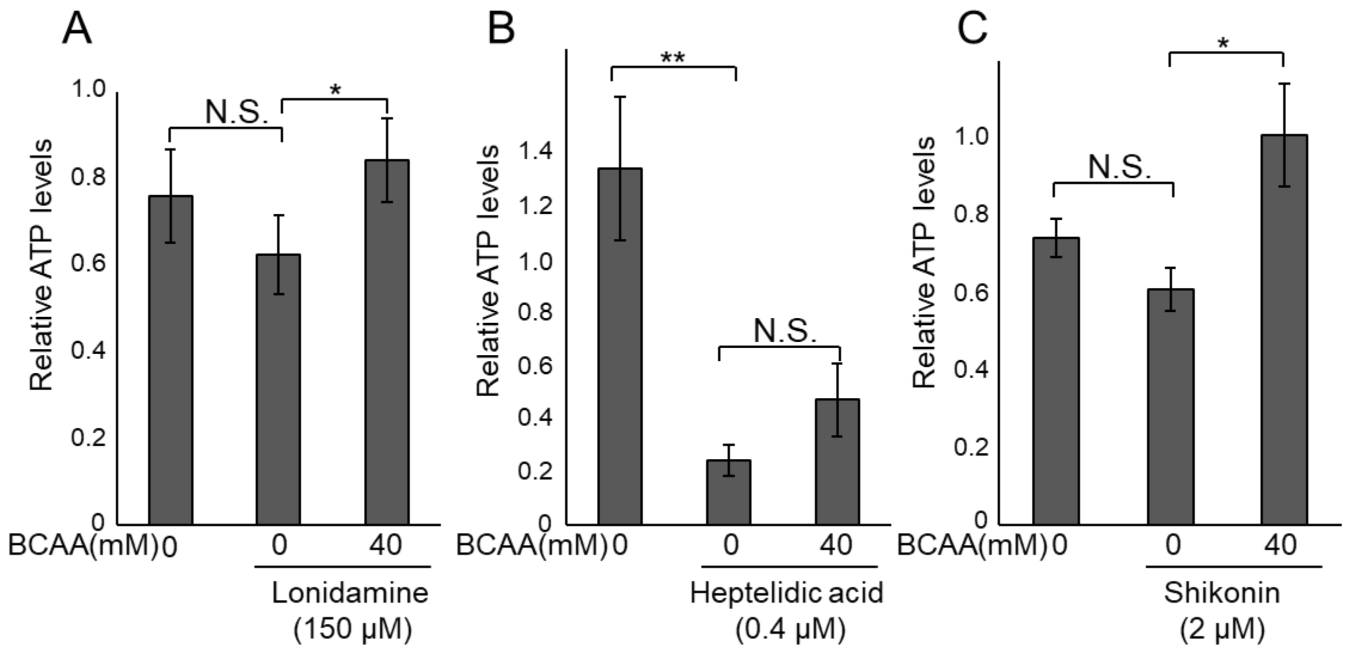

Supple Figure S1. Recovery of intracellular ATP levels in cultured cells by branched chain amino acids (BCAAs). HeLa cells were cultured in amino acid-free medium containing 4.5 g/L glucose with or without 40 mM BCAAs and with or without 150 μM lonidamine (A), 0.4 μM heptelidic acid (B), or 2 μM shikonin (C). After HeLa cells were cultured for 24 h in (A) and (C) or 48 h in (B), relative intracellular ATP levels were determined using a luciferase assay. *p < 0.05, **p < 0.01, N.S. no significant difference, Tukey honestly significant difference (HSD), N = 4. Bars represent standard deviation.

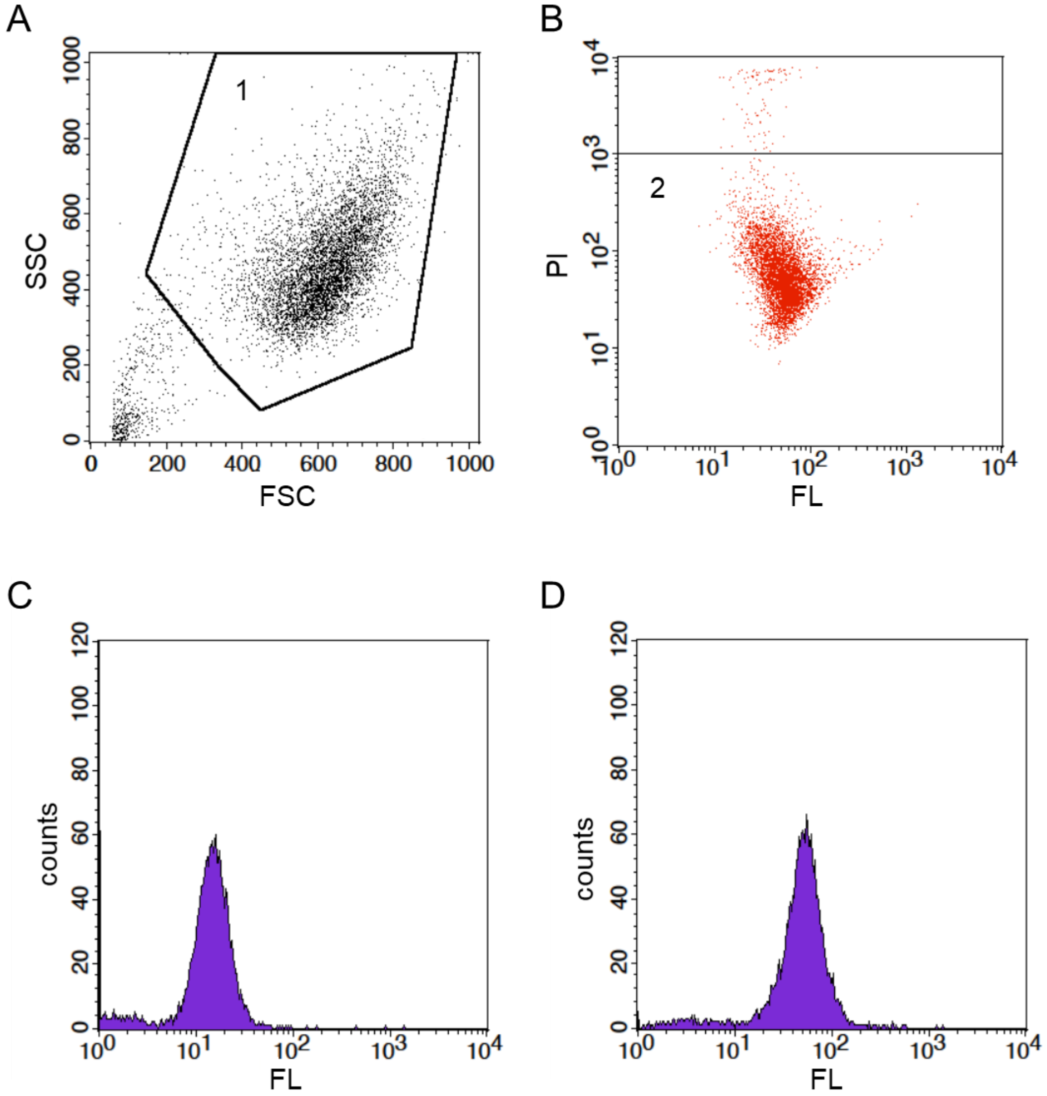

Supple Figure S2. Analysis of viable cells and glucose uptake by flow cytometry. Cells were incubated with (D) or without (C) fluorescently labeled deoxy-glucose analog (2-NBDG) and propidium iodide (PI). (A) Analysis of cells by forward scatter (FSC)-area (x-axis) and side scatter (SSC)-area (y-axis). The subpopulation in area 1, which excluded subpopulations including dregs with small FSC and SSC, was regarded as cells. (B) The subpopulation in area 1 was analyzed by fluorescein fluorescence (FL, x-axis) and PI fluorescence (PI, y-axis). Cells in area 2 without high PI fluorescence were regarded as live cells. Live cell ratios were calculated as the ratio of the counts in the subpopulation in area 2 to the subpopulation in area 1. (C, D) Live cells, a subpopulation in area 2, were analyzed using fluorescein fluorescence (FL, x-axis). Cells without 2-NBDG showed low fluorescein fluorescence (C, negative control), while cells incubated with 2-NBDG showed high fluorescein fluorescence (D).

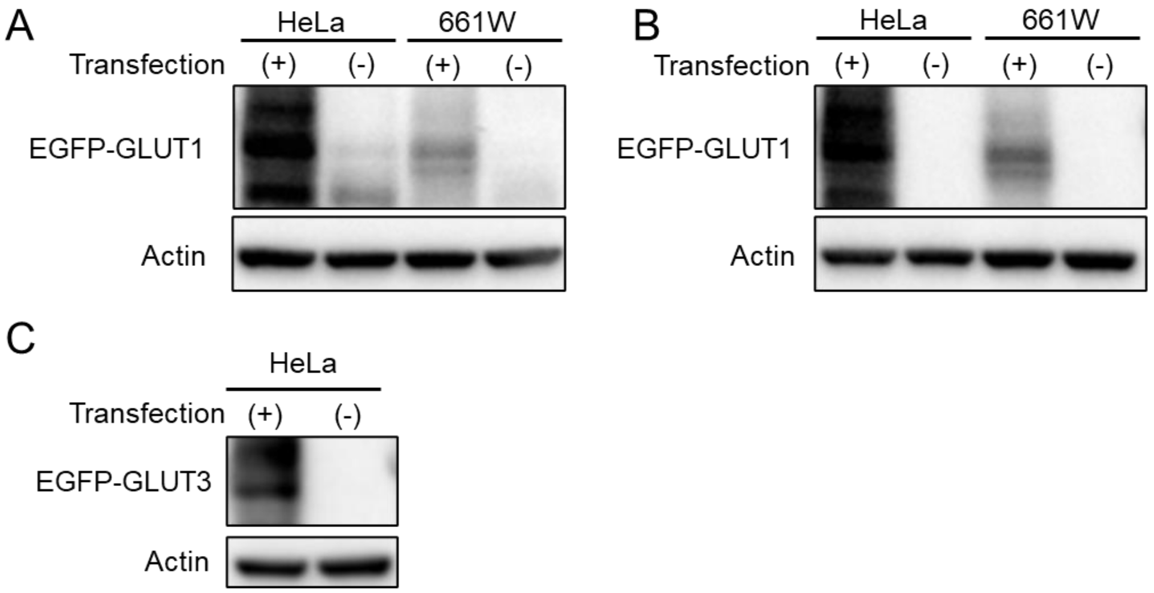

Supple Figure S3. Enhanced green fluorescent protein (EGFP)-GLUT1 or GLUT3 expression in HeLa and 661W cells. Cells transfected with vectors carrying EGFP-GLUT1 or GLUT3 (Transfection (+)) and cells without transfection (Transfection (-)) as a control were analyzed by western blotting. (A, B) EGFP-GLUT1 expression was analyzed using an anti-GLUT1 antibody (A, Abcam, ab115730) or an anti-GFP antibody (B, MBL, 598). (C) EGFP fusion-GLUT3 expression was analyzed using an anti-GFP antibody. Actin was used as loading control.

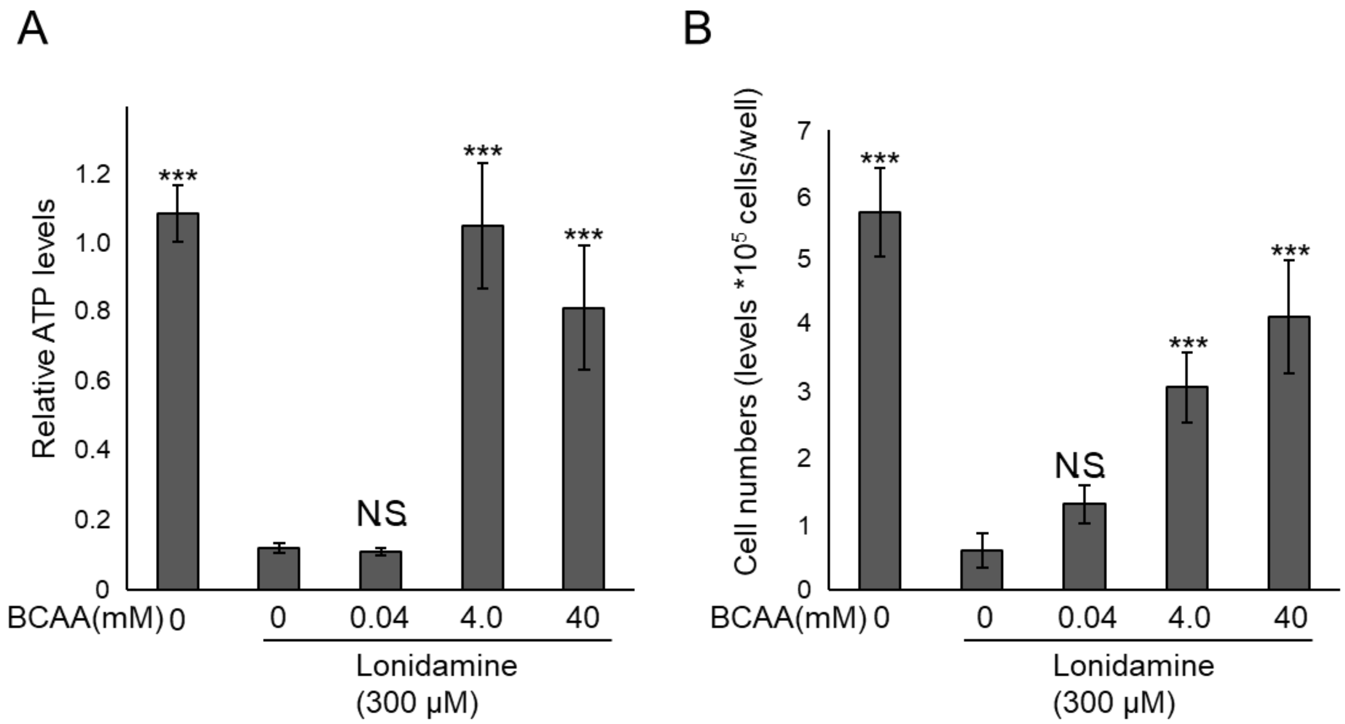

Supple Figure S4. Recovery of intracellular ATP levels and prevention of cell death by BCAAs under glycolysis inhibition. HeLa cells were cultured in amino acid-free medium containing 4.5 g/L glucose with or without BCAAs (0, 0.04, 4.0, or 40 mM) and with or without 300 μM lonidamine. After the HeLa cells were cultured for 24 h, the relative intracellular ATP levels were determined using a luciferase assay (A), and live cell numbers were measured after trypsinization with a TC20 cell counter (Bio-Rad) (B). ***$p < 0.005$, N.S. no significant difference, vs with lonidamine without BCAAs, Tukey HSD, N = 4, respectively. Bars represent standard deviation.

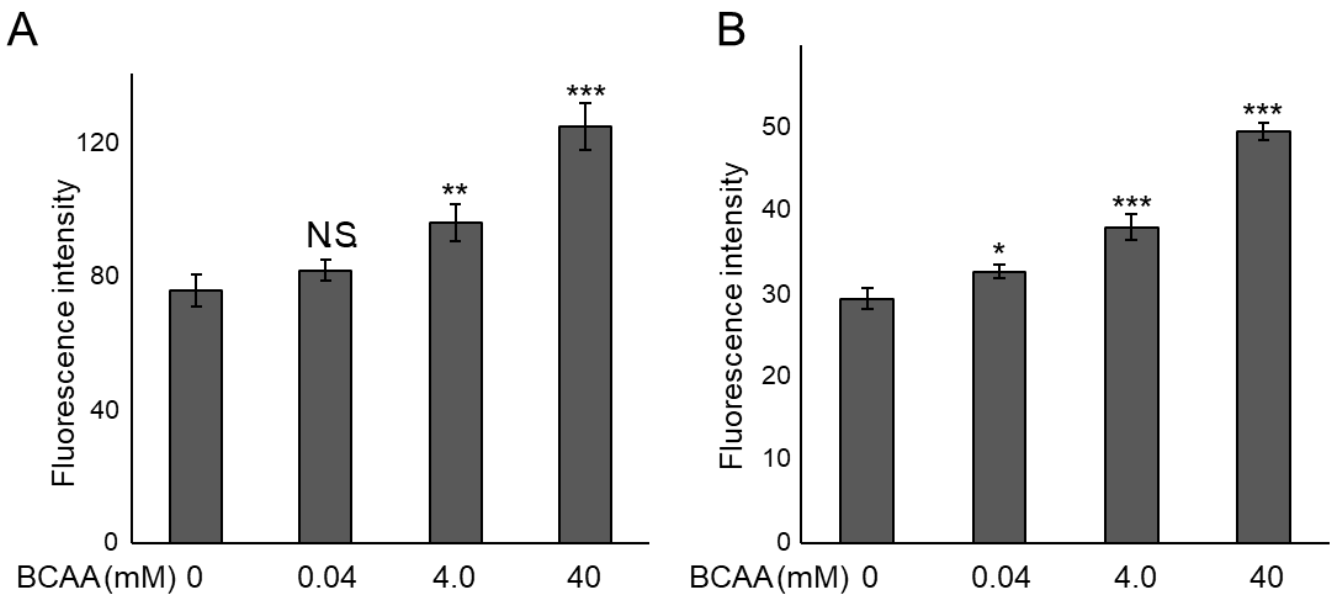

Supple Figure S5. Promotion of glucose uptake in cultured cells by BCAAs at various concentrations. HeLa cells (A) and 661W cells (B) were cultured in amino acid-free medium containing 4.5 g/L glucose with or without BCAAs (0.04, 4.0, or 40 mM) for 24 h before adding fluorescently labeled deoxy-glucose analog (100 μM 2-NBDG). (A, B) Geometric average of fluorescence intensities of live cells analyzed by flow cytometry analysis. *$p < 0.05$, **$p < 0.01$, ***$p < 0.0001$, N.S. no significant difference, vs without BCAAs, Tukey HSD, N = 4. Bars represent standard deviation

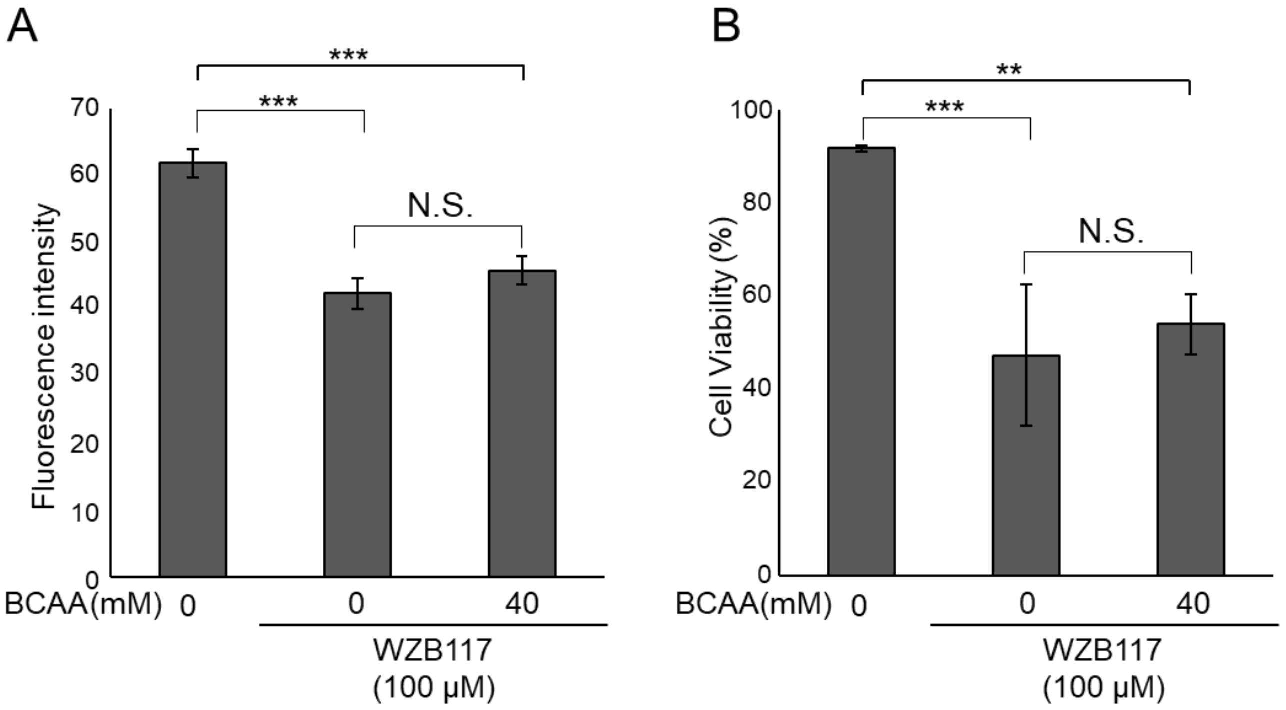

Supple Figure S6. Glucose uptake and cell viability with or without BCAAs under glucose transporter inhibition. HeLa cells were cultured in amino acid-free medium containing 4.5 g/L glucose with or without 40 mM BCAAs for 3 h and then incubated with or without 100 μM WZB117 for 1 h before adding fluorescently labeled deoxy-glucose analog (100 μM 2-NBDG). (A) Glucose uptake shown as geometric average of fluorescence intensities of live cells analyzed by flow cytometry. (B) Cell viability. **$p < 0.01$, ***$p < 0.0001$, N.S. no significant difference, Tukey HSD, N = 4 each. Bars represent standard deviation.

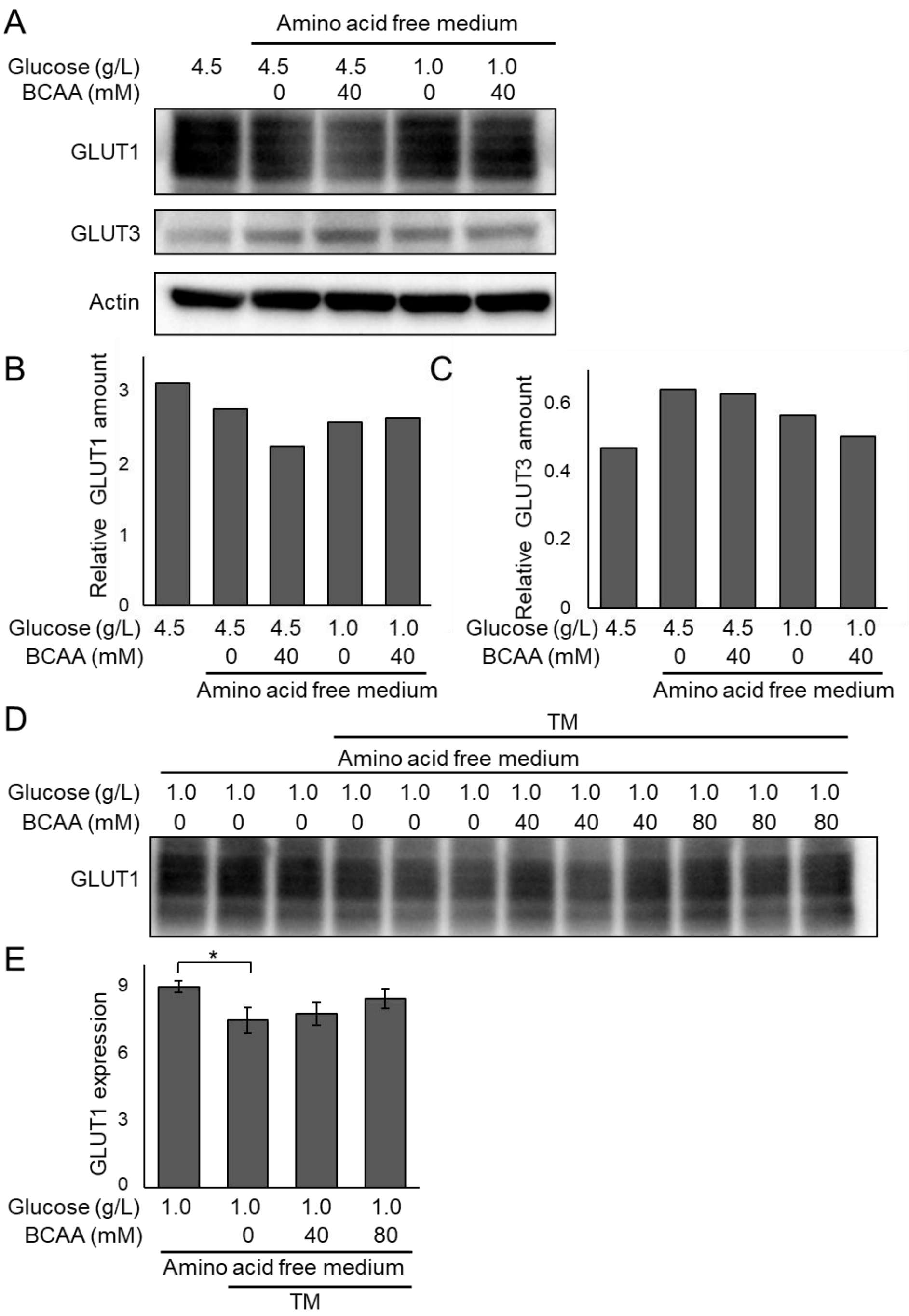

Supple Figure S7. Glucose transporter (GLUT) expression in HeLa cells. HeLa cells were cultured in DMEM containing 1.0 or 4.5 g/L of glucose without amino acids, with or without BCAAs (40 mM or 80 mM) and with or without 3 μg/mL tunicamycin (TM) for 24 h (A–C) or 6 h (D, E). (A–C) GLUT1 and GLUT3 expression in whole cells was analyzed using anti-GLUT1 antibody (Abcam, ab115730) or anti-GLUT3 antibody (Abcam, ab41525). (B, C) Relative expression of GLUT1 and GLUT3; Actin was used as loading control. (D, E) Plasma membrane fractions were extracted from HeLa cells using a Plasma Membrane Protein Extraction Kit (101 Bio), and GLUT1 expression was analyzed. (E) GLUT1 expression. *$p < 0.05$, Turkey HSD, N = 3. Bars represent standard deviation.

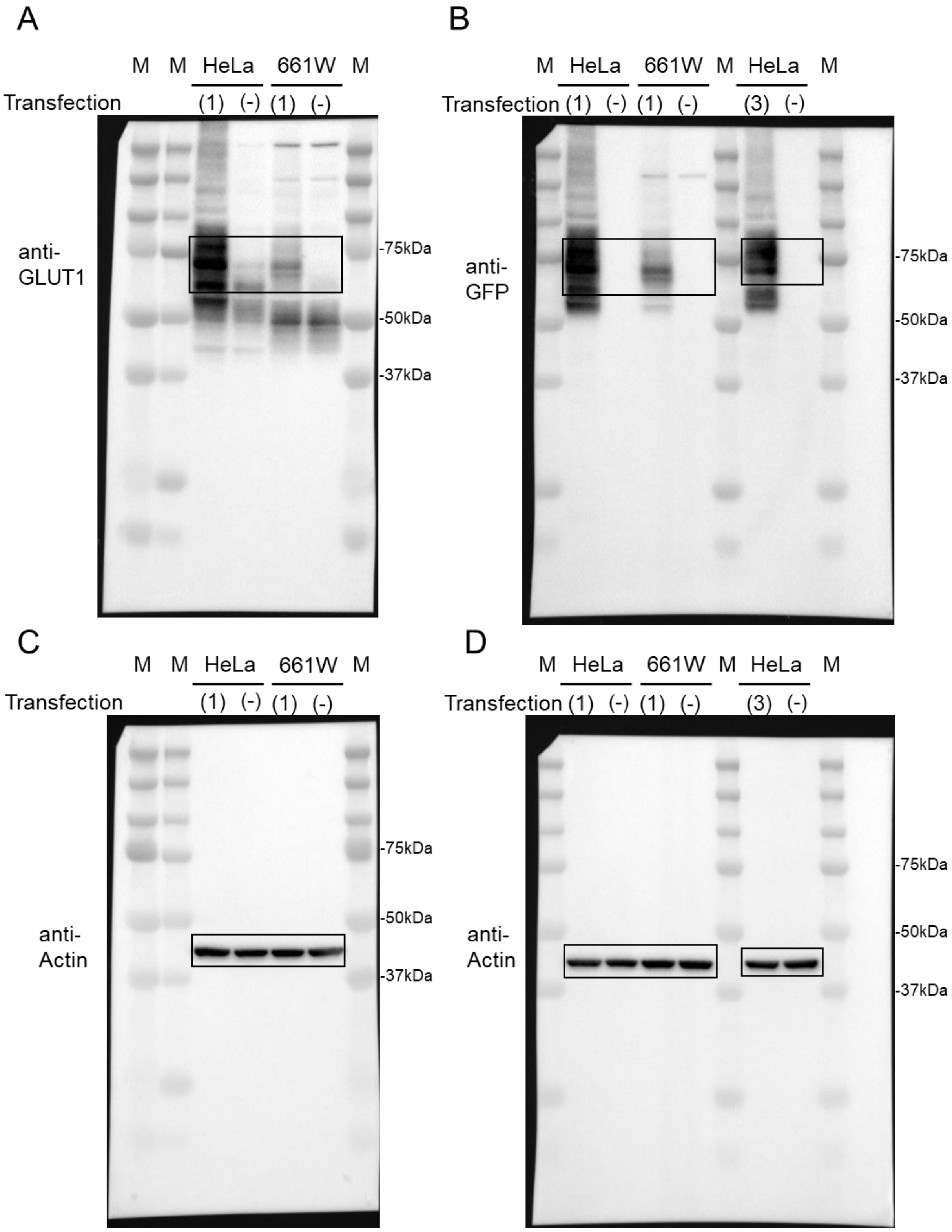

Supple Figure S8. Complete scans of western blots related to Supplementary Figure S3. HeLa cells and 661 W cells transfected with vectors carrying EGFP-GLUT1 (Transfection (1)) or GLUT3 (Transfection (3)) and cells without transfection (Transfection (-)) were analyzed by western blotting. EGFP-GLUT1 was analyzed using an anti-GLUT1 antibody (A), and EGFP-GLUT3 was analyzed using an anti-GFP antibody (B). Actin was used as a loading control (C, D). M: Molecular weight marker.

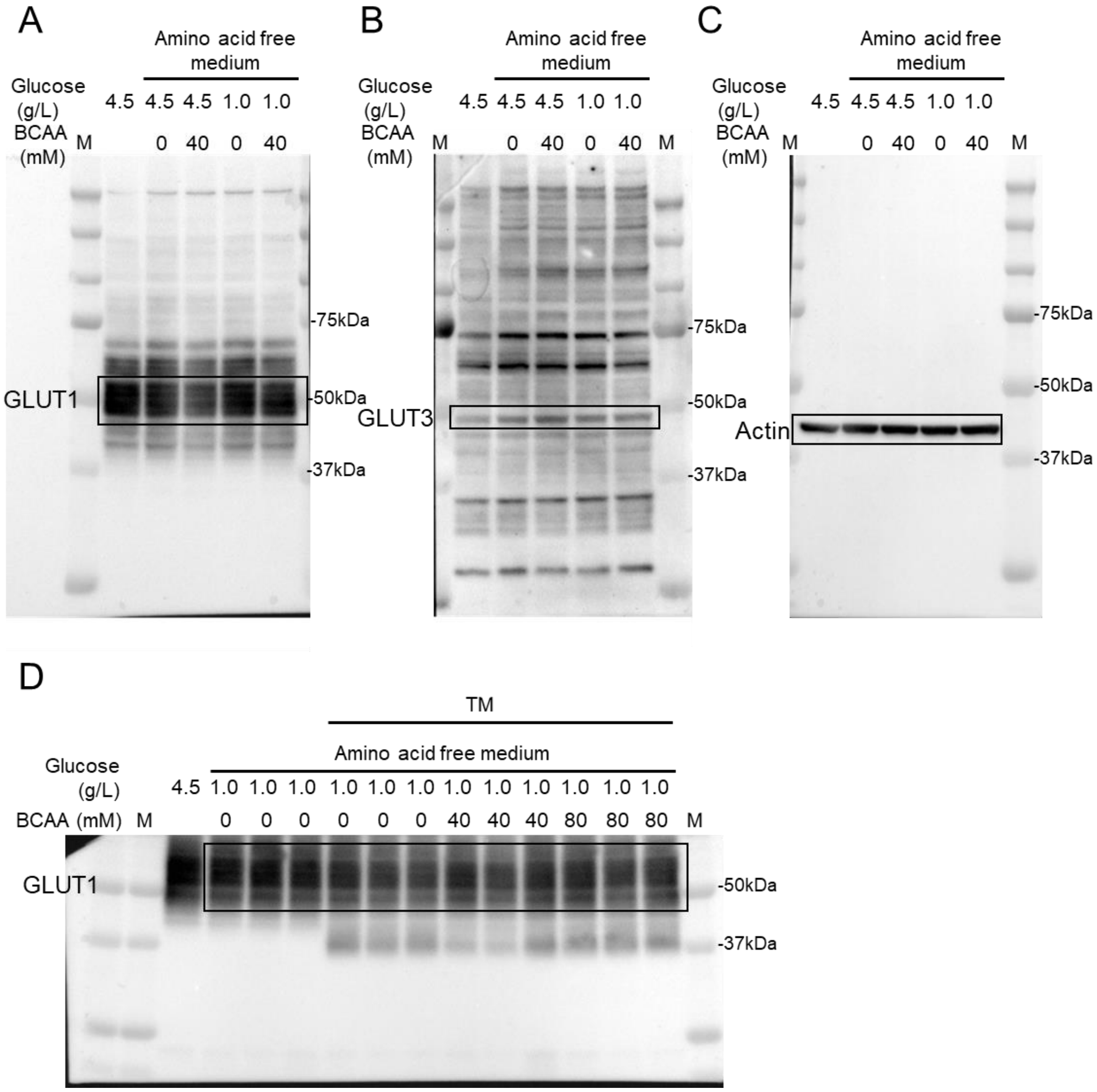

Supple Figure S9. Complete scans of western blots related to Supplementary Figure S7. HeLa cells were cultured in DMEM containing 4.5 or 1.0 g/L of glucose with or without amino acids, with or without BCAAs (40 mM or 80 mM) and with or without tunicamycin 3 μg/mL (TM). GLUT1 (A, D) and GLUT3 (B) expression in whole cells (A, B) or in the plasma membrane fraction (D) was analyzed. Actin was used as loading control (C). M: Molecular weight marker.
